# Computational Identification of Natural Inhibitors Targeting Fiber Proteins of FAdV-1 and FAdV-4 Through Integrated Virtual Screening and Molecular Dynamics Simulations

**DOI:** 10.3390/vetsci13030223

**Published:** 2026-02-26

**Authors:** Amina Kardoudi, Salaheddine Redouane, Abdelouaheb Benani, Faouzi Kichou, Charifa Drissi Touzani, Siham Fellahi

**Affiliations:** 1 Department of Veterinary Pathology and Public Health, Hassan II Agronomic and Veterinary Institute, B.P. 6202, Rabat 10000, Morocco; f.kichou@iav.ac.ma (F.K.); charifadrissi@gmail.com (C.D.T.); s.fellahi@iav.ac.ma (S.F.); 2 Molecular Biology Laboratory, Medical Biology Department, Pasteur Institute of Morocco, Casablanca 20000, Morocco; abdelouaheb.benani@pasteur.ma; 3 Laboratory of Genomics and Human Genetics, Institute Pasteur du Maroc, Casablanca 10000, Morocco; eredouane95@gmail.com

**Keywords:** fowl adenovirus, FAdV-1, FAdV-4, fiber protein, ANPDB, SANCDB, virtual screening, molecular docking, ADMET, molecular dynamic simulation

## Abstract

Fowl adenoviruses (FAdVs) represent a major threat to poultry health, with serotypes FAdV-1 and FAdV-4 causing adenoviral gizzard erosion (AGE) and hepatitis-hydropericardium syndrome (HHS), respectively. A wide variety of afflicted birds, including chicken, pigeon, and psittacine species, have been reported to carry aviadenoviruses. The disease is highly contagious and spreads rapidly between flocks and farms through vertical and horizontal transmission. In this study, we implemented a multi-stage computational drug-discovery pipeline to identify natural inhibitors of the viral fiber proteins for both FAdV-1 and FAdV-4. A curated library of 7523 natural compounds from the African Natural Products Database (ANPDB) and the South African Natural Compounds Database (SANCDB) was subjected to ADMET-based filtering, molecular docking, ADMET prediction, and 500 ns molecular dynamics simulations against four structural targets: Fiber-1 and Fiber-2 of FAdV-4, and the Short and Long Fibers of FAdV-1. Three ligands, ANPDB_6449 (−10.3 kcal/mol), ANPDB_2908 (−10.2 and −10.0 kcal/mol), and SANCDB_245 (−9.2 kcal/mol), consistently emerged as strong candidates across the entire computational workflow. While ANPDB_2908 demonstrated notable multi-target capability by binding to fiber proteins from both FAdV-1 and FAdV-4, ANPDB_6449 and SANCDB_245 exhibited strong serotype-specific potential, supported by stable interaction profiles and favorable drug-likeness characteristics. Together, these compounds highlight promising natural scaffolds for the development of targeted antiviral interventions against pathogenic FAdV serotypes.

## Simple Summary

Viral diseases in poultry cause major economic losses worldwide and threaten food production. Two important viruses, known to damage the liver and digestive organs of chickens, spread rapidly between farms and are difficult to control. Current prevention methods mainly rely on vaccination and farm hygiene, but there are no specific antiviral treatments available if birds become infected. This study aimed to discover natural compounds that could potentially block key viral proteins responsible for the virus attaching to and entering chicken cells. Using advanced computer-based methods, we screened thousands of plant-derived molecules from African natural product collections to identify those most likely to bind strongly to these viral proteins. The most promising compounds were further evaluated for their stability, safety, and behavior in biological systems using predictive models. Several natural molecules showed strong and stable interactions with the viral targets and displayed characteristics suggesting they could be safe for further testing. While these findings are based on computer simulations and must still be confirmed in laboratory and animal studies, they provide an important first step toward developing new antiviral strategies for poultry. Such treatments could help reduce disease spread, limit economic losses for farmers, and contribute to more secure and sustainable poultry production worldwide.

## 1. Introduction

Fowl adenoviruses (FAdVs) belong to the *Adenoviridae* family and the *Aviadenovirus* genus, encompassing avian adenoviruses that share a common antigen. To date, five FAdV species (FAdV-A to FAdV-E) have been identified by complete genome analysis using restriction endonucleases, while the viral neutralization (VN) test has identified 12 serotypes (1–7, 8a, 8b, 9–1) [1,2]. Due to their vertical and horizontal transmission, FAdVs are widespread throughout the world [3] and are associated with significant economic losses in the poultry industry. Although most infections are subclinical, some FAdV serotypes are associated with impactful poultry diseases such as inclusion body hepatitis (IBH) [3,4], adenoviral gizzard erosion (AGE) [5,6], pancreatic necrosis [7,8], and hepatitis-hydropericardium syndrome (HHS) [8,9].

Hepatitis-hydropericardium syndrome, primarily associated with FAdV serotype 4, is characterized by sudden onset of high mortality, accumulation of straw-colored fluid in the pericardial sac, and severe hepatic necrosis. Mortality rates in affected flocks can range from 30% to 80%, especially in young broilers aged 3 to 6 weeks [10,11]. FAdV-4 associated with HHS has been reported in Pakistan [12], India [13], Japan [14], China, Iran [15], Egypt [16], South Korea [17], Iraq [18], and Saudi Arabia [19], highlighting the wide geographic distribution of the disease and its growing significance in global poultry production.

Adenoviral gizzard erosion is mainly caused by fowl adenovirus serotype 1 (FAdV-1) [6,20], although other serotypes such as FAdV-4, -8a, and -8b have occasionally been isolated from affected birds [18,19]. The disease is characterized by erosion and ulceration of the gizzard’s koilin layer, often with hemorrhage and sloughing of the mucosa. Although AGE usually causes low mortality, it leads to poor growth, and reduced feed efficiency, making it economically important [17,21,22,23]. Initially described in Japan, AGE has since been reported in many other countries. In Asia, cases have been reported in South Korea [24], China [25], Malaysia [26], Iran [27], and India [28]. In Europe, the disease has been found in Italy [29], Sweden [30], Poland [31], Hungary [32], Belgium [33], Slovakia [34], and the United Kingdom [23]. AGE has also been reported in Egypt [35] and Morocco [7]. This wide distribution highlights the global spread of disease and the importance of monitoring FAdV infections in poultry farms.

*Aviadenoviruses* have been found in a wide range of infected bird species, including chickens and pigeons [36,37]. Infection in psittacine species is caused by aviadenovirus [38,39]. In Australia, an adenovirus-like illness was discovered in peach-faced lovebirds and a cockatiel. Depression, anorexia, diarrhea, and cloacal bleeding have all been linked to adenovirus infections in psittacine birds.

For serotype 4, Fiber-1 has recently been identified as a key determinant of viral entry, specifically interacting with the Coxsackievirus and Adenovirus Receptor (CAR) through its knob domain, which is essential for viral attachment and penetration into host cells [39]. In addition, the Fiber-2 protein has been widely demonstrated as a major virulence factor in several recent studies [40]. Reports have shown that the high pathogenicity of emerging FAdV-4 strains is closely associated with the fiber-2 gene. Furthermore, subunit vaccines based on the Fiber-2 protein have provided effective protection against FAdV-4 infection, and monoclonal antibodies targeting Fiber-2 have been shown to efficiently block infection and reduce viral pathogenicity [41]. Together, Fiber-1 and Fiber-2 represent complementary and strategic targets for FAdV-4 inhibition [42].

Regarding serotype 1, it is important to note that this virus possesses two distinct fiber proteins, known as the Short Fiber (Fiber-2) and the Long Fiber (Fiber-1); both of them play crucial roles in its pathogenicity and infectivity [43]. Studies have highlighted the cooperative involvement of these fibers in the viral infection cycle, justifying their selection as potential targets for antiviral intervention. More recent studies have clarified the molecular determinants of this interaction, demonstrating that Fiber-1 mediates CAR binding in both FAdV-1 and FAdV-4 through its knob domain, which specifically engages domain 1 (D1) of the CAR [44].This receptor–ligand interaction has been directly visualized using confocal microscopy, confirming Fiber-1 as the primary determinant of CAR-dependent viral entry in these serotypes [45].

Natural products remain an abundant source of bioactive compounds. For centuries, traditional medicine has relied on plant extracts to treat various diseases, leading to the identification of numerous phytochemicals with anticancer, antidiabetic, antioxidant, and anti-inflammatory activities [46,47,48]. Technological advances have enabled the isolation and characterization of these compounds, reaffirming their central role in drug discovery and ethnopharmacology [49,50].

In this context, virtual screening provides an efficient approach to exploring large libraries of natural compounds and identifying molecules capable of interacting with specific viral targets. Molecular docking predicts interactions between target proteins and potential inhibitors, while molecular dynamics simulations allow evaluation of the stability and behavior of these complexes over time. Together, they provide valuable insights into receptor–ligand interactions and are now considered standard approaches in modern drug discovery [51,52].

The main objective of this study is to identify potential inhibitors targeting the Fiber-1 and Fiber-2 proteins of FAdV-4 and the Short and Long Fiber proteins of FAdV-1 using a virtual screening approach. These proteins play crucial roles in viral attachment and infectivity, making them promising targets for the development of specific antiviral agents.

## 2. Materials and Methods

### 2.1. Databasess Preparation

We performed virtual screenings with compounds contained in the African Natural Products Database (ANPDB) (https://phabidb.vm.uni-freiburg.de/anpdb/; accessed on 16 February 2026) and South African Natural Compounds Database (SANCDB) (https://sancdb.rubi.ru.ac.za/; accessed on 16 February 2026). It is important to note that the ANPDB contains information on compounds from different parts of Africa [53,54,55] specifically, it provides three main folders; one that contains compounds from only north African regions, a second one that contains only compounds from east African regions and a third one that contains compounds from both north and east African regions together. The third folder was the only one chosen from ANPDB with a total of 6511 compounds and the first two databases wereexcludedto removeduplicates entries, whereas SANCDB is dedicated to natural compounds collected from South Africa regions and contains a total of 1,012 compounds [49,50]. Altogether, a total of 7523 natural African compounds were retrieved from the databases [56]. Thereafter, the compounds were downloaded in “Sdf” format protonated and subjected to a 2500-step MMFF94 energy minimization using Open Banel 3.1.1. Subsequently, they were converted to PDBQT format for molecular docking analyses using the same software.

### 2.2. Proteins Preparation

The crystal structures of the fiber proteins from FAdV-1 and FAdV-4 were retrieved from the Protein Data Bank (PDB)**.** The corresponding PDB IDs were as follows: *FAdV-4 Fiber-1 (PDB ID: 7X5T), FAdV-4 Fiber-2 (PDB ID: 7W83), FAdV-1 Long Fiber (PDB ID: 2IUM), and FAdV-1 Short Fiber (PDB ID: 2VTW)*. To facilitate the presentation of the results, the Fiber-1 and Fiber-2 proteins of FAdV-4 are consistently referred to as Fiber-1 and Fiber-2 throughout the manuscript. In contrast, for FAdV-1, the Short Fiber is occasionally labeled as Fiber-2 and the Long Fiber as Fiber-1, particularly in Section 3.3.

Prior to molecular docking, all protein structures were prepared using online tools such as PDBsum (https://www.ebi.ac.uk/pdbsum/; accessed on: 20 February 2026), PrankWeb (https://prankweb.cz/; accessed on: 20 February 2026), and tools installed on local machine as PyMol (https://pymol.org; accessed on 16 February 2026) and AutoDockTools (https://autodock.scripps.edu; accessed on 16 February 2026). PDBsum was used to identify experimentally derived structural clefts and surface features of the fiber proteins, helping to guide the initial localization of potential ligand-binding regions [57], while PrankWeb, a machine-learning-based ligand-binding site predictor, was employed to identify probable ligandable pockets based on structural and physicochemical features of the protein surface [58]. The structural analysis focused on the homotrimeric forms of Fiber-1/Long Fiber and Fiber-2/Short Fiber from fowl adenovirus serotypes 4 and 1, using 2IUM and 2VTW for serotype 1 and 7X5T and 7W83 for serotype 4, consistent with the homotrimeric organization described by [59]. For each structure, both mmCIF and PDB formats were retrieved: the mmCIF files were specifically chosen because they contain the complete homotrimeric assemblies essential for analyzing the natural oligomeric architecture of each fiber, while the PDB monomeric files were used in parallel to understand how each trimeric unit is organized at the monomer–monomer interface (Supplementary Data S1). Across all serotypes and fibers, the docking pockets were identified using a combined PDBsum-PrankWeb strategy that integrates experimentally defined clefts with probabilistic ligandability predictions. For serotype 4 Fiber-2 (7W83), although a previous study by Chandramohan [60] docked ligands near residues ASP413, ARG313, and ARG377 without structural justification, PrankWeb Pocket 1 composed of residues 385, 387, 389, 406, 407, 408, 429, 430, 431, 471, 472, 473, and 474 was independently validated and selected (Supplementary Data S1, Figure S1). All structures were refined in PyMOL by removing crystallographic waters/non-essential heteroatoms and incorporating PDBsum-generated scripts to visualize clefts and confirm predicted pockets, and the specific targeting for molecular docking was carried out using AutoDock Tools of MGLTools 1.5.6 package, by positioning the grid box precisely on the predefined pockets identified for each fiber protein. For serotype 1 (2IUM, 2VTW) and serotype 4 Fiber-1 (7X5T), the absence of experimentally confirmed receptor-binding sites required applying the same systematic PDBsum-PrankWeb workflow (Supplementary Data S1, Figures S2–S4). This comprehensive multi-target approach was done on refined Fibers’ structures and energetically minimized using steepest descent approach with GROMACS 2021.3 software, which enabled the identification of biologically relevant receptor-binding pockets suitable for docking natural antiviral phytochemicals from ANPDB and SANCDB.

### 2.3. Molecular Docking

Virtual screening of retrieved natural compounds against the fiber surface proteins of FAdV-4 and FAdV-1 was carried out using AutoDock Vina; accessed on 16 February 2026. Prior to docking, the three-dimensional structures of the target proteins were prepared using AutoDock Tools, where polar hydrogen atoms and Kollman charges were added to the protein chains to generate the docking-ready PDBQT files. All default docking parameters were maintained. The grid box was defined with a spacing of 0.375 Å, centered at the active site coordinates determined from the binding pocket of each target, with a grid size of 52 Å × 50 Å × 48 Å, centered at x = 30.302, y = 34.868, z = 32.999 for Long Fiber (2IUM) of serotype 1 (Supplementary Data S1, Figure S4c) and 85 Å × 64 Å × 70 Å, centered at x = 49.428, y = 18.402, z = 33.200 for Short Fiber (2VTW) of serotype 1 (Supplementary Data S1, Figure S3c) and 36 Å × 34 Å × 58 Å, centered at x = 29.711, y = 29.966, z = 44.278 for fiber 1 (7X5T) of serotype 4 (Supplementary Data S1, Figure S2c) and 76 Å × 72 Å × 74 Å, centered at x = −2.067, y = −17.794, z = −15.406 for fiber 2 (7W83) of serotype 4 (Supplementary Data S1, Figure S1c).

All prepared PDBQT files corresponding to the 7523 natural compounds were subjected to structure-based virtual screening (SBVS) through protein–ligand molecular docking. Nine docking poses were generated for each ligand and ranked according to their binding affinities. The results were visualized and analyzed using Discovery Studio Visualizer v17.2 and PyMOL v1.1 to identify key molecular interactions within the active site [51,59]. The top-ranked complexes showing the lowest docking energies and most favorable hydrogen-bond and hydrophobic interactions were selected for subsequent molecular dynamics simulations [61,62,63].

Following molecular docking, the selected compounds were organized into four groups to facilitate comparative analysis and interpretation of the docking results. Group 1 corresponded to the docking results between ligands and the Fiber-1 protein of FAdV-4, Group 2 to the Fiber-2 protein of FAdV-4, Group 3 to the Short Fiber protein of FAdV-1, and Group 4 to the Long Fiber protein of FAdV-1. This classification enabled a systematic evaluation of binding affinities and interaction patterns within and between serotypes. From each group, the ten compounds exhibiting the lowest binding energies were selected as the most promising candidates for further analysis. These top-ranked ligands were subsequently subjected to a detailed ligand–protein interaction analysis using Discovery Studio Visualizer. Only molecules forming the most relevant and structurally meaningful interactions including stable hydrogen bonds, hydrophobic contacts, and π-related interactions within the predicted binding pockets were retained for the next stage of the study. Because no experimentally validated small-molecule inhibitors of FAdV fiber proteins are currently available, classical redocking validation was not feasible. Instead, docking reliability was supported by the consistent localization of top-ranked poses within the same predicted pockets and the agreement between independent pocket prediction methods (PDBsum and PrankWeb).

### 2.4. Pharmacokinetic Properties and Drug-likeness

The top-ranked ligands from each group were subsequently subjected to Absorption, Distribution, Metabolism, Excretion, and Toxicity (ADMET) analysis to evaluate their pharmacokinetic and toxicological properties. The ADMET screening was carried out using ADMETlab 3.0 (https://admetlab3.scbdd.com/, accessed on 20 January 2026) and ProTox-3 (https://tox.charite.de/protox3/, accessed on 20 January 2026). ADMETlab 3.0 was used to assess parameters such as oral bioavailability, metabolic stability, and excretory behavior [64,65,66], while ProTox-3 predicted hepatotoxicity, mutagenicity, carcinogenicity, and organ-specific toxicities [63,64]. Compounds meeting standard drug-likeness criteria including compliance with Lipinski’s Rule of Five, absence of mutagenic and hepatotoxic effects, and acceptable pharmacokinetic profiles were retained as the most promising candidates for further in silico validation targeting the Fiber proteins of FAdV-1 and FAdV-4.

### 2.5. Molecular Dynamic Simulation

Molecular dynamics simulations were performed using GROMACS 2021.3 [65,66] software and charmm27.ff force fields for the protein combined with a parametrization of each ligand/natural compound using SwissParam online tool; accessed on 16 February 2026, at combines Merck Molecular ForceField (MMFF) and multipurpose atom-typer for CHARMM (MATCH) approach [67,68,69]. The SPC/E water model was selected due to its stable thermodynamic properties and its common use in CHARMM-based biomolecular simulations, ensuring reliable solvation behavior. To investigate the structural stability and dynamic behavior of the selected ligand-protein complexes identified from molecular docking, he simulations were carried out for 500 ns to evaluate the interaction stability between the top-ranked natural compounds and their respective target proteins (Fiber-1 and Fiber-2 of FAdV-4, and the Short and Long Fibers of FAdV-1). Each complex was solvated in a triclinic water box using the SPC/E water model, and counterions (Na^+^/Cl^−^) were added to neutralize the system and mimic physiological conditions. Long-range electrostatic interactions were calculated using the Particle Mesh Ewald (PME) method with a real-space cutoff of 1.2 nm. Van der Waals interactions were also truncated at 1.2 nm using a force-switching function. Energy minimization was realized to eliminate steric clashes and reduce potential energy. Two equilibration steps were conducted prior to production: an NVT ensemble to stabilize temperature (at 310 K) with fixed seeding (gen_seed = 123,456) and an NPT ensemble to stabilize pressure (at 1 bar). Each equilibration phase (NVT and NPT) was conducted for 500 ps before the production run. Temperature was controlled using the V-rescale (modified Berendsen) thermostat, while pressure was maintained using the Parrinello-Rahman barostat. The production phase of the simulation was performed for 500 ns with a 2 fs time step, saving coordinate frames every 10 ps for subsequent analyses. All covalent bonds involving hydrogen atoms were constrained using the LINCS algorithm, allowing the use of a 2 fs integration time step. Post-simulation trajectory analyses included the calculation of the Root Mean Square Deviation (RMSD) of both target protein (fiber) and the bounded ligand (natural compound) to assess conformational stability, the Root Mean Square Fluctuation (RMSF) of the target protein to evaluate residue flexibility, and the Radius of Gyration (Rg) of the target protein to determine the overall compactness of the complexes. The Hydrogen Bonds number (Hbond) between each target protein and its bounded ligand wasused to investigate the interaction stability profiles, and Principal Component Analysis (PCA) and its derived Gibbs free energy landscapes (FEL) were analyzed for overall dynamics behavior of each target protein’s backbone. These plots were generated as xvg files using respectively these commands: gmx rms, gmx rmsf, gmx gyrate, gmx hbond, gmx covar, gmx anaeig, gmx sham and gmx xpm2ps. Each parameter xvg files’ group was visualized using QtGrace tool. No replica simulations were performed, which is acknowledged as a limitation of the study.

## 3. Results

### 3.1. Virtual Screening and Molecular Docking

**Figure 1:**
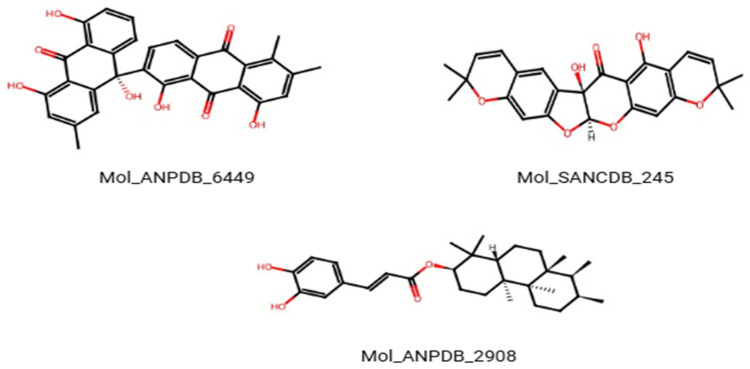
Two-dimensional structures of the top three natural ligands (ANPDB_6449, SANCDB_245, ANPDB_2908) selected based on docking affinity and ADMET profiling.

Computer-aided drug discovery (CADD) relies heavily on molecular docking as a core technique for evaluating the interaction between a receptor and a large number of candidate compounds. This approach uses computational algorithms to estimate binding affinity, interaction energies, and ligand–receptor contacts within a virtual environment. In the present study, molecular docking was employed to identify potential inhibitors targeting the fiber proteins of two important FAdV serotypes: FAdV-1 (Short Fiber and Long Fiber) and FAdV-4 (Fiber-1 and Fiber-2). A total of 7523 natural compounds derived from African medicinal plants were collected from two curated databases and docked against each of the four target proteins. All compounds were ranked in ascending order based on their docking scores and compared with the previously reported ligand dehydroepiandrosterone (DHEA), which exhibited a binding affinity of −7.7 kcal/mol for FAdV-4 Fiber-2. From the 7523 screened molecules, one top-scoring ligand per target was selected, each showing the lowest binding energy within its respective dataset. The complete list of screened compounds and their binding affinities is provided in Supplementary Data S2, ordered from the lowest to the highest docking energy for the four targets (Figure 1).

Table 1 provides an in-depth overview of how the highest-scoring ligands interact with each fiber protein. Ligands displaying the lowest docking energies and advantageous interaction characteristics are deemed the most promising. Interestingly, the ligand ANPDB_2908 exhibited notably high binding affinity for both the Long Fiber of FAdV-1 and Fiber-2 of FAdV-4.

The interaction analysis of the four top-ranked ligands revealed distinct but consistently favorable binding behaviors across the different FAdV fiber proteins. For the Long Fiber of FAdV-1, ANPDB_6449 displayed a high binding affinity of −10.3 kcal/mol, forming three conventional hydrogen bonds with Thr704 and engaging in stabilizing hydrophobic interactions with Thr632 and Tyr705, along with additional van der Waals contacts involving Pro604, Ala657, and Val541, collectively ensuring a well-anchored conformation within the binding cavity. A similar trend of strong polar-driven binding was observed for the Short Fiber of FAdV-1, where ANPDB_2908 achieved an affinity of −10.2 kcal/mol by forming two hydrogen bonds with Asn344 and Trp350, complemented by ancillary interactions with Ala348, indicating a stable and favorable insertion into the Long Fiber pocket.

For the Fiber-1 of FAdV-4, SANCDB_245 exhibited a balanced and well-distributed interaction profile, with a binding affinity of −9.2 kcal/mol. The ligand established three hydrogen bonds with Asp320 and Arg344, supported by hydrophobic contributions from Tyr323 and Gly324, and additional stabilizing contacts with Pro341 and Gly322, reflecting a strong compatibility with the structural and chemical properties of the Fiber-1 binding site.

Extending this pattern, the ligand ANPDB_2908 also showed a remarkable affinity for FAdV-4 Fiber-2 (−10.0 kcal/mol), where it formed three conventional hydrogen bonds with Val270, Asn273, and Tyr328, supported by hydrophobic interactions with Ala330. This combination of polar and non-polar contacts suggests a cooperative stabilization mechanism in which hydrogen bonding, complemented by hydrophobic packing, drives a robust and energetically favorable binding mode.

### 3.2. Drug Metabolism and Toxicity Profiling

In this study, we employed in silico ADMET prediction tools to assess the pharmacokinetic and toxicological profiles of the three top-scoring natural compounds identified through virtual screening against FAdV fiber proteins. The predicted absorption, distribution, metabolism, excretion, and toxicity parameters for these ligands are summarized in Table 2.

The absorption profile showed moderate Caco-2 permeability values for all compounds, while Human Intestinal Absorption (HIA) predictions were not available in the ADMETlab output. For distribution, none of the compounds were predicted to cross the blood–brain barrier, and the volume of distribution (VD) values suggested moderate systemic distribution. High plasma protein binding (PPB > 92%) was observed for all ligands, indicating strong binding to serum proteins.

The metabolism assessment, based on five cytochrome P450 isoforms (CYP1A2, CYP2C19, CYP2C9, CYP2D6, and CYP3A4), revealed distinct inhibitory patterns among the ligands. ANPDB_6449 and SANCDB_245 showed strong predicted inhibition of CYP2C9 (“+++”), whereas ANPDB_2908 displayed no CYP2C9 inhibition. For the other CYP isoforms, ANPDB_6449 showed weak inhibition of CYP1A2 and CYP2D6, while SANCDB_245 exhibited minor inhibition toward CYP2C19. ANPDB_2908 demonstrated minimal predicted interaction with the tested CYP enzymes. The excretion profile, evaluated through total clearance and half-life values, indicated moderate clearance rates and relatively short half-lives, suggesting limited accumulation in the body. Toxicity predictions showed low probabilities of hERG channel inhibition for all compounds, indicating a low predicted risk of cardiotoxicity.

Overall, these ADMET predictions suggest acceptable pharmacokinetic properties, although potential CYP2C9 inhibition should be considered in future experimental validation.

### 3.3. Molecular Dynamic Simulation

To further investigate the stability and conformational behavior of the ligand–protein interactions over time, molecular dynamics (MD) simulations were performed for the four selected complexes: ANPDB_6449-Long Fiber (FAdV-1), ANPDB_2908-Short Fiber (FAdV-1), SANCDB_245-Fiber-1 (FAdV-4), and ANPDB_2908-Fiber-2 (FAdV-4). Each complex was simulated for 500 ns using GROMACS to assess how ligand binding evolves dynamically and to characterize the structural fluctuations and stability of the interaction.

#### 3.3.1. Root Mean Square Deviation

Root Mean Square Deviation is a widely used parameter in molecular dynamics to monitor the structural stability of protein–ligand complexes over time. It reflects how much the backbone of the targeted protein deviates from its initial conformation during the simulation. Lower and stable RMSD values generally indicate a well-equilibrated system and stable ligand binding, whereas large fluctuations may suggest structural rearrangements or unstable interactions.

**Figure 2:**
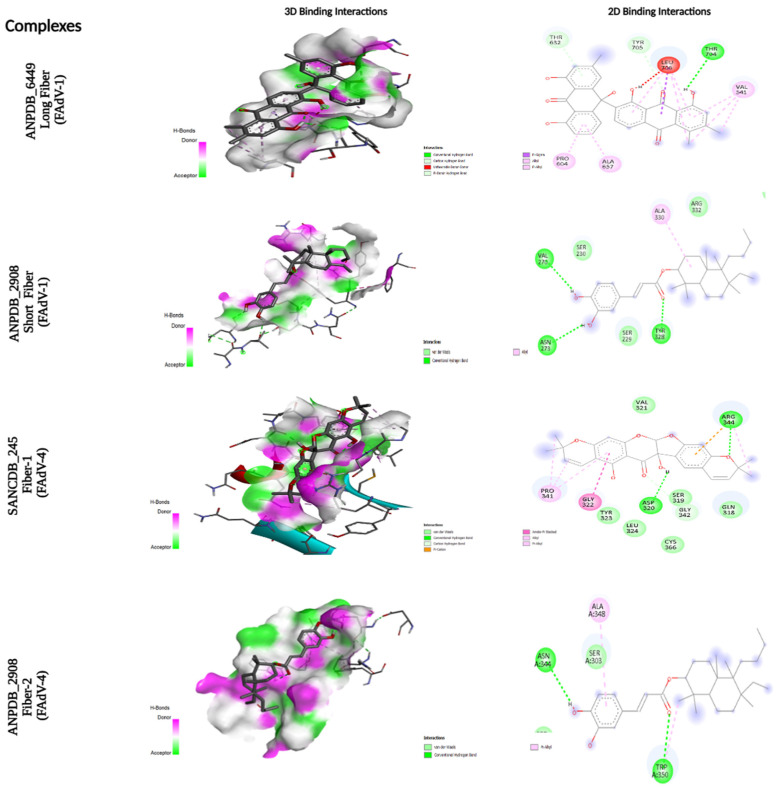
Three-dimensional (3D) and two-dimensional (2D) binding interaction profiles of the top-ranked natural compounds docked against four FAdV fiber proteins. From top to bottom: ANPDB_6449 bound to the Long Fiber of FAdV-1, ANPDB_2908 bound to the Short Fiber of FAdV-1, SANCDB_245 interacting with Fiber-1 of FAdV-4, and ANPDB_2908 bound to Fiber-2 of FAdV-4. The 3D interaction maps illustrate the spatial orientation of each ligand within the binding pocket, highlighting hydrogen bond donor (magenta) and acceptor (green) regions, as well as hydrophobic surfaces. Key interacting amino acid residues are shown within the binding environment. The 2D interaction diagrams detail the specific ligand–protein contacts, including conventional hydrogen bonds, carbon–hydrogen bonds, hydrophobic (alkyl and π–alkyl) interactions, and π–π stacking where present. Interacting residues are labeled with their corresponding amino acid names and residue numbers, allowing precise identification of binding site contacts.

**Figure 3:**
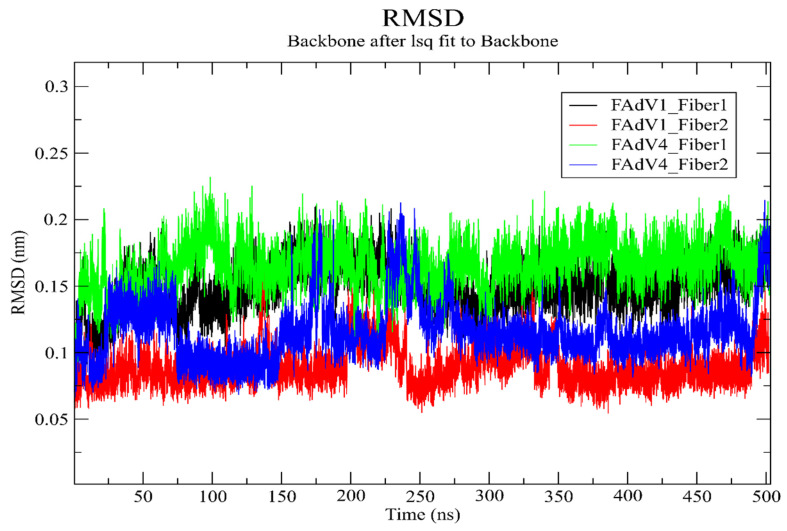
Root Mean Square Deviation profiles of the four ligand–fiber protein complexes (FAdV-1 Fiber-1, FAdV-1 Fiber-2, FAdV-4 Fiber-1, and FAdV-4 Fiber-2) over a 500 ns molecular dynamics simulation, showing the backbone stability and global conformational fluctuations of each complex.

In this study, the RMSD profiles of the four simulated complexes, including FAdV1 (Short and Long Fiber), FAdV4-Fiber1, and FAdV4-Fiber2, were examined over a 500 ns trajectory to evaluate their conformational behavior and binding stability (Figure 2 and Figure 3). The RMSD plot shows that all four complexes reached equilibrium early in the simulation and maintained relatively stable fluctuations throughout the 500 ns run, indicating that ligand binding remained stable across all systems. Among the complexes, FAdV1-Fiber2 displayed the lowest and most stable RMSD values, consistently oscillating around 0.07-0.10 nm, suggesting a very rigid and stable interaction with its ligand. FAdV4-Fiber2 also exhibited a stable profile, fluctuating slightly higher around 0.10–0.15 nm, which still reflects good structural stability. The FAdV1-Fiber1 complex showed moderate fluctuations between 0.12 and 0.17 nm but maintained a steady overall trend, indicating acceptable conformational equilibrium. The highest RMSD values were observed for the FAdV4-Fiber1 complex, which fluctuated around 0.15–0.22 nm, reflecting a more flexible backbone yet still remaining within a stable range for protein–ligand systems.

Figure 1: Root Mean Square Deviation profiles of the four ligand–fiber protein complexes (FAdV-1 Fiber-1, FAdV-1 Fiber-2, FAdV-4 Fiber-1, and FAdV-4 Fiber-2) over a 500 ns molecular dynamics simulation, showing the backbone stability and global conformational fluctuations of each complex.

#### 3.3.2. Root Mean Square Fluctuation

Root Mean Square Fluctuation provides residue-level insights into the flexibility of protein regions during the simulation, where higher RMSF values indicate more mobile or unstable segments and lower values reflect rigid, stable regions. Across all four complexes, the RMSF profiles show overall low fluctuations, indicating that ligand binding maintains the structural stability of each fiber protein.

**Figure 4:**
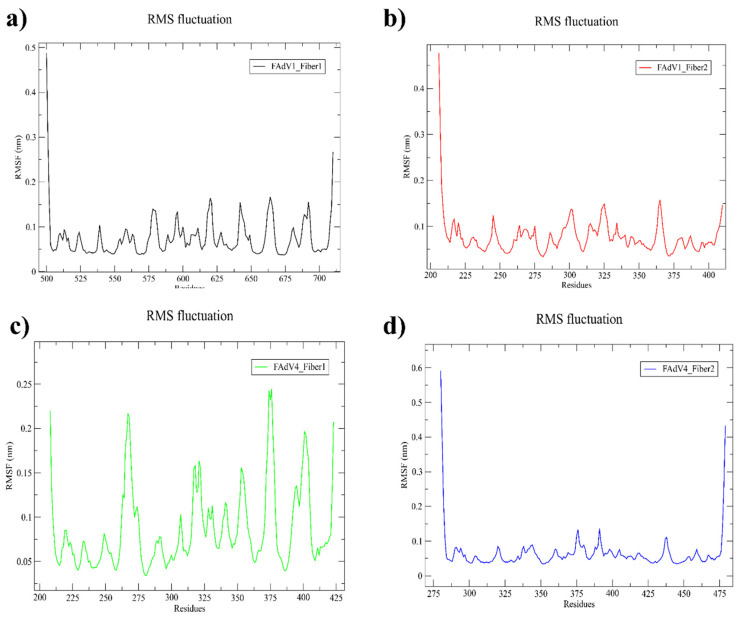
Root Mean Square Fluctuation analysis of backbone residues for the four ligand–fiber protein complexes ((**a**). FAdV-1 Fiber-1, (**b**). FAdV-1 Fiber-2, (**c**). FAdV-4 Fiber-1, and (**d**). FAdV-4 Fiber-2), highlighting residue-level flexibility and local dynamic variations during the 500 ns simulation.

For FAdV-1 Fiber-1 (a), fluctuations remain minimal throughout the chain with only slight increases in loop regions, reflecting a globally rigid backbone. FAdV-1 Fiber-2 (b) shows a similar pattern, with low RMSF values along most residues and a higher peak at the N-terminus, consistent with terminal flexibility. In FAdV-4 Fiber-1 (c), moderate fluctuations appear at exposed loops around residues 270–310 and 360–400, but the overall profile remains within a stable range. FAdV-4 Fiber-2 (d) displays slightly higher terminal mobility compared to the other complexes, while internal residues show uniformly low fluctuations, indicating stable ligand–protein interactions (Figure 4).

#### 3.3.3. Two-Dimensional Principal Component Projection

**Figure 5:**
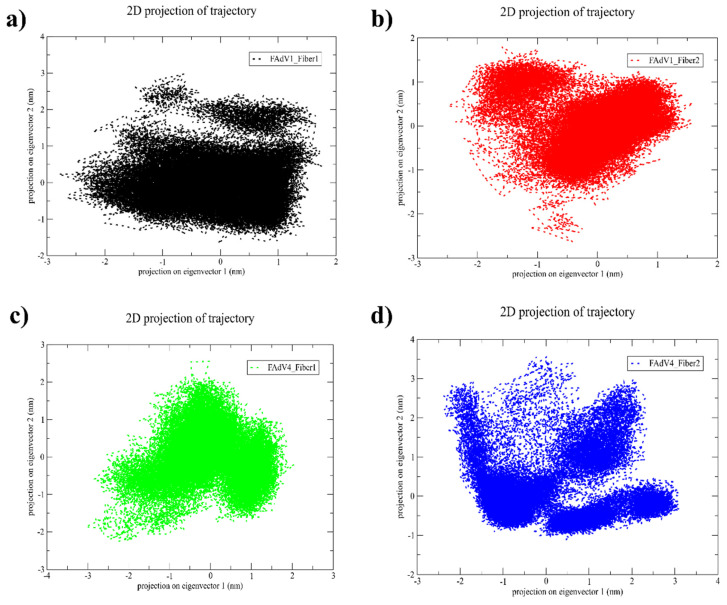
Two-dimensional (2D) PCA projection of the 500-ns MD trajectories for the four ligand–fiber complexes, showing conformational sampling along the first two principal components: (**a**) FAdV-1 Fiber-1, (**b**) FAdV-1 Fiber-2, (**c**) FAdV-4 Fiber-1, and (**d**) FAdV-4 Fiber-2.

Principal Component Analysis was performed to characterize the dominant conformational motions sampled by each ligand–fiber complex throughout the 500 ns simulation (Figure 5). The 2D projections along the first two principal components (PC1 and PC2) revealed distinct distributions of conformational states for the four systems. The FAdV-1 Fiber-1 complex displayed a compact distribution, suggesting restricted structural fluctuations and stable sampling of a narrow conformational basin. In contrast, the FAdV-1 Fiber-2 complex presented a broader and more dispersed trajectory, indicating higher flexibility and a larger conformational landscape. The FAdV-4 Fiber-1 complex exhibited an intermediate pattern with moderate spreading along PC1 and PC2, consistent with a dynamic but structurally controlled system. The FAdV-4 Fiber-2 complex showed the widest dispersion among the four trajectories, reflecting substantial conformational diversity and multiple low-energy substates. Overall, PCA demonstrates that each ligand–fiber system explores a characteristic conformational space, with FAdV-1 Fiber-1 being the most structurally restricted and FAdV-4 Fiber-2 the most flexible during the simulation.

#### 3.3.4. Gibbs Free Energy Landscape Analysis

The Gibbs free energy landscapes derived from the PCA projections provide insight into the conformational stability and energetic preferences explored by each ligand–fiber complex during the molecular dynamic simulations. These landscapes map the distribution of conformational states along the first two principal components (PC1 and PC2), where low-energy basins (blue) represent the most stable conformations sampled by the system, and high-energy regions (yellow to red) indicate less favorable and sparsely populated states.

**Figure 6:**
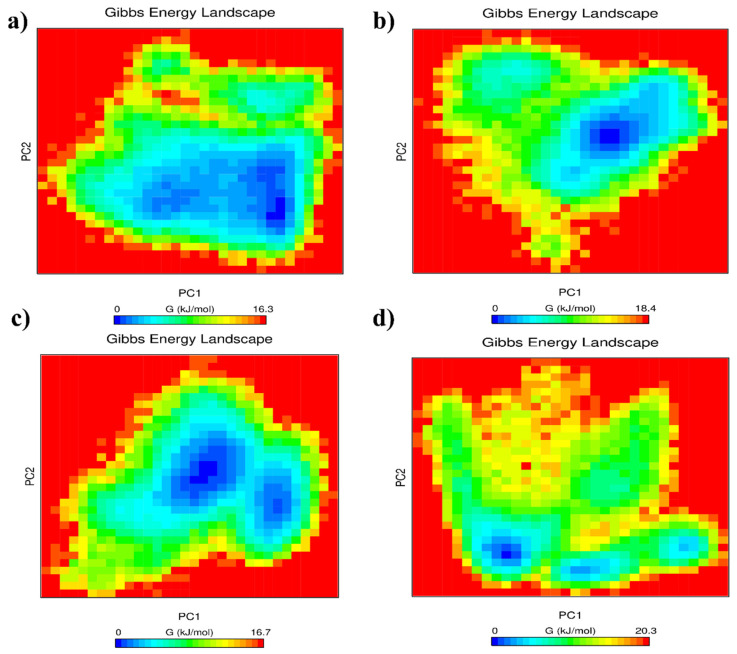
Gibbs free energy landscapes of the four ligand–fiber complexes projected onto the first two principal components (PC1 and PC2): (**a**) FAdV-1 Fiber-1, (**b**) FAdV-1 Fiber-2, (**c**) FAdV-4 Fiber-1, and (**d**) FAdV-4 Fiber-2. Low-energy basins (blue) indicate stable conformational states, whereas higher-energy regions (yellow to red) represent less favorable conformations.

For the FAdV-1 Fiber-1 complex (Figure 6a), the landscape shows a broad and deep low-energy basin, indicating that the complex remains confined within a relatively stable conformational substate throughout the simulation. The absence of multiple deep minima suggests limited conformational transitions and a well-stabilized binding mode. The FAdV-1 Fiber-2 complex (Figure 6b) exhibits a more segmented low-energy region, with two partially connected minima. This indicates that the complex samples at least two favorable conformations during the trajectory, reflecting a moderate degree of structural flexibility within the binding pocket while maintaining overall energetic stability. In the case of FAdV-4 Fiber-1 (Figure 6c), the landscape displays a well-defined low-energy basin surrounded by narrow intermediate-energy zones, suggesting stable binding accompanied by minor fluctuations in local conformational substates. This pattern is consistent with a ligand comfortably accommodated in the binding cavity but experiencing slight reorientation over the simulation course. Finally, the FAdV-4 Fiber-2 complex (Figure 6d) presents a more diverse and extended free energy topology, characterized by multiple interconnected minima. This reflects higher conformational mobility and indicates that the system explores a wider range of energetically accessible states, suggesting dynamic interactions between the ligand and the fiber protein.

Across the four ligand–fiber complexes, the Gibbs free energy landscapes revealed clear differences in conformational stability. The Short Fiber-ANPDB_6449 and Fiber-1-SANCDB_245 complexes displayed the most compact and deep energy basins, indicating stable conformational states with limited energetic fluctuations. In contrast, the Long Fiber-ANPDB_2908 and Fiber-2-ANPDB_2908 complexes showed broader and more diffuse basins, suggesting greater conformational flexibility and a wider range of accessible states. Overall, the landscapes highlight that the ligands binding to Short Fiber and Fiber-1 adopt more energetically favorable and stable conformations compared with those binding to Long Fiber and Fiber-2.

## 4. Discussion

Fowl adenoviruses continue to pose a major economic threat to global poultry production, particularly serotypes FAdV-1 and FAdV-4, which are responsible for adenoviral gizzard erosion and hepatitis-hydropericardium syndrome, respectively. While AGE is generally associated with low mortality but significant production losses [6], HHS remains one of the most severe adenoviral diseases in broilers, with mortality rates frequently ranging from 30% to 80% [9,70,71,72,73]. The growing number of outbreaks reported worldwide underscores the urgent need for novel therapeutic strategies targeting the molecular determinants of these viruses [71]. In this context, bioinformatics-driven drug discovery has emerged as a powerful approach, offering the ability to screen large natural compound libraries and to characterize ligand–protein interactions with high precision [72]. In the present study, we employed a comprehensive virtual screening approach using two natural compound libraries (ANPDB and SANCDB), followed by molecular docking, detailed protein–ligand interaction analysis, and pharmacokinetic profiling; and long-timescale molecular dynamics simulations were then integrated to evaluate the ability of these natural compounds to inhibit the fiber proteins of both FAdV-1 and FAdV-4, which play essential roles in viral attachment and pathogenicity. The following discussion contextualizes and interprets the computational results obtained for each of the selected ligand–protein complexes.

Across all analyses, three ligands, ANPDB_6449, ANPDB_2908, and SANCDB_245, demonstrated superior binding energies, structural compatibility, and dynamic stability compared with previously reported antiviral candidates such as dehydroepiandrosterone (DHEA), whose binding affinity to FAdV-4 Fiber-2 was −7.7 kcal/mol [57]. Although DHEA has been suggested as a potential inhibitor of FAdV fiber proteins, its steroidal nature raises concerns regarding specificity and safety due to its endocrine activity, which could limit its use in poultry [73,74]. In contrast, natural products offer greater structural diversity and biological compatibility, along with the potential to act on multiple viral or host targets. These findings reinforce the potential of African natural compounds as valuable scaffolds for antiviral drug development.

ANPDB_6449 displayed the strongest affinity for the Long Fiber of FAdV-1 (−10.3 kcal/mol). Its interaction pattern included three hydrogen bonds with Thr704, supplemented by hydrophobic contacts with Thr632 and Tyr705, and alkyl interactions with Pro604, Ala657, and Val541, collectively anchoring the ligand in a stable conformation. Despite the presence of an unfavorable interaction with Leu706, the global stability of the complex was not compromised.

Regarding metabolism, ANPDB_6449 showed a selective inhibitory profile toward CYP enzymes. The ligand is predicted to inhibit CYP1A2 and strongly inhibit CYP2C9, while exhibiting no inhibitory activity toward CYP2C19, CYP2D6, or CYP3A4. This pattern suggests that the molecule is unlikely to undergo extensive biotransformation through most major CYP isoenzymes, with CYP2C9 being the primary metabolic interaction site. Such selectivity may help reduce broad metabolic interference while indicating a specific pathway for potential drug–drug interactions.

During the 500 ns MD simulation, the complex maintained low RMSD values (0.12–0.20 nm) and minimal residue fluctuations, supporting a rigid and stable ligand–fiber interface. PCA and Gibbs free energy analyses further revealed a restricted conformational landscape with a single dominant energy basin, indicating strong thermodynamic stability. Together, these results suggest that ANPDB_6449 constitutes a promising candidate for targeting AGE-associated FAdV-1 strains.

ANPDB_2908 demonstrated high affinity for two different viral proteins: the Short Fiber of FAdV-1 and the Fiber-2 protein of FAdV-4, highlighting its multi-target potential. For the Long Fiber, ANPDB_2908 interacted through two key hydrogen bonds with Asn344 and Trp350, complemented by hydrophobic contacts with Ala348. ADMET analyses indicated excellent intestinal absorption, absence of CYP inhibition, and overall favorable pharmacokinetic behavior. The MD simulation revealed the lowest RMSD fluctuations among the four complexes (0.07–0.10 nm), indicating a highly stable binding mode. PCA showed moderate conformational variability, while the Gibbs free energy surface exhibited two interconnected low-energy minima, reflecting dynamic but energetically stable binding. These results emphasize the suitability of ANPDB_2908 as a potent inhibitor of Short Fiber-mediated viral entry mechanisms.

Remarkably, ANPDB_2908 also displayed strong inhibitory potential against FAdV-4Fiber-2, with a binding affinity of −10.0 kcal/mol, outperforming DHEA (−7.7 kcal/mol) [60]. Its binding was stabilized by hydrogen bonds with Val270, Asn273, and Tyr328 and reinforced by hydrophobic contacts with Ala330. MD simulations showed low RMSD values (0.10–0.15 nm) and consistently reduced RMSF levels for core residues. PCA demonstrated broad conformational sampling consistent with the intrinsic flexibility of Fiber-2, while the Gibbs free energy map displayed interconnected minima characteristic of adaptive yet stable ligand accommodation. These results demonstrate that ANPDB_2908 efficiently adapts to the dynamic Fiber-2 pocket, offering superior stability compared with previously documented antiviral molecules.

SANCDB_245, selected for Fiber-1 of FAdV-4, exhibited a docking affinity of −9.2 kcal/mol and formed three hydrogen bonds with Asp320 and Arg344, in addition to stabilizing hydrophobic and amide-π interactions with Tyr323, Gly324, Pro341, and Gly322. ADMET profiles highlighted good absorption and limited metabolic inhibition, primarily involving CYP2C9. During the MD simulation, RMSD values (0.15-0.22 nm) confirmed long-term structural stability, while RMSF variations were restricted to exposed loop regions characteristic of Fiber-1 flexibility. PCA indicated moderate conformational sampling, and the Gibbs landscape exhibited a stable single-basin architecture, confirming the structural compatibility of SANCDB_245 with the Fiber-1 pocket.

When comparing the four complexes, ANPDB_6449 (for FAdV-1 Long Fiber) and SANCDB_245 (for FAdV-4 Fiber-1) exhibited the deepest, most compact energy basins, indicative of highly stable interactions. Conversely, the ANPDB_2908 complexes (Short Fiber and Fiber-2) explored wider conformational landscapes while maintaining stability, reflecting the adaptability of this ligand to structurally diverse adenoviral receptors. Unlike DHEA, which showed limited interaction networks and moderate MD stability in previous studies, the natural compounds identified here demonstrated stronger affinities, more robust interaction patterns, and enhanced dynamic stability.

Collectively, the integrated computational results highlight ANPDB_2908 as the most promising broad-spectrum candidate, capable of targeting key proteins from both FAdV-1 and FAdV-4. Meanwhile, ANPDB_6449 and SANCDB_245 represent strong serotype-specific inhibitors for the Long Fiber of FAdV-1 and Fiber-1 of FAdV-4, respectively. These findings underscore the potential of African natural compounds as scaffolds for future antiviral development and provide a foundation for subsequent in vitro and in vivo validation studies aimed at controlling both AGE- and HHS-associated adenoviral infections.

## 5. Conclusions and Perspective

This study identified three promising natural compounds, ANPDB_6449, ANPDB_2908, and SANCDB_245, as potential inhibitors of key fiber proteins involved in the pathogenicity of FAdV-1 and FAdV-4. Through integrated virtual screening, ADMET profiling, and 500 ns molecular dynamics simulations, these ligands demonstrated strong binding affinities, favorable pharmacokinetics, and stable interaction dynamics. Among them, ANPDB_2908 emerged as a broad-spectrum candidate targeting both serotypes, while ANPDB_6449 and SANCDB_245 showed strong serotype-specific inhibition. These findings provide a solid computational basis for future in vitro validation and the development of targeted antiviral strategies against AGE- and HHS-associated FAdV infections. Although the present findings are based exclusively on computational analyses, they offer a strong rationale for subsequent experimental validation. Future studies should therefore assess the antiviral activity and cytotoxicity of these compounds in vitro, followed by in vivo evaluation in relevant poultry infection models to confirm their potential as candidates and support the development of antiviral interventions against FAdV-associated diseases.

## Figures and Tables

**Table 1:** Docking scores and interaction profiles of selected ligands with FAdV fiber proteins.

Ligands	Target Protein	Binding Affinity	Residues Involved in Conventional Hydrogen Bond Formation	Number of Hydrogen Bonds Formed	Residues Involved in Hydrophobic Interactions	Residues Involved in Other Interactions
Mol_ANPDB_6449	Long Fiber FAdV-1	−10.3	Thr704	3	Thr632, Tyr705	Pro604, Ala657, VAL541
Mol_ANPDB_2908	Short Fiber FAdV-1	−10.2	Asn344, Trp350	2	0	Ala348
Mol_SANCDB_245	Fiber-1 FAdV-4	−9.2	Asp320, Arg344	3	Try323, Gly324	Pro341, Gly322
Mol_ANPDB_2908	Fiber-2 FAdV-4	−10	Val270, Asn273, Tyr328	0	0	Ala330

**Table 2:** In silico ADMET predictions, including absorption, distribution, metabolism, excretion, and toxicity parameters, for the four lead compounds selected from virtual screening.

Property	Model Name	ANPDB_6449	ANPDB_2908	SANCDB_245
Absorption	Caco-2 Permeability (cm/s)	−5.228	−5.191	−5.02
	HIA	---	---	---
Distribution	BBB penetration (cm/s)	-	-	-
	PPB (%)	97.0	92.7	96.5
	VD (L/Kg)	0.667	0.174	0.295
Metabolism	CYP1A2 inhibitor	+	---	---
	CYP2C19 inhibitor	---	---	-
	CYP2C9 inhibitor	+++	---	+++
	CYP2D6 inhibitor	--	---	---
	CYP3A4 inhibitor	-	---	---
Excretion	CL plasma(mL/min/Kg)	2.02	15.265	5.692
	T1/2 (H)	1.815	0.999	1.546
Toxicity	hERG blockers	0.012	0.063	0.169

## Data Availability

The original contributions presented in this study are included in the article/Supplementary Material. Further inquiries can be directed to the corresponding author.

## Supplementary Materials

The following supporting information can be downloaded at: https://www.mdpi.com/article/10.3390/vetsci13030223/s1, Data S1: Supplementary data S1; Data S2: Supplementary data S2.
