# Supplementary material for: Computational Identification of Natural Inhibitors Targeting Fiber Proteins of FAdV-1 and FAdV-4 Through Integrated Virtual Screening and Molecular Dynamics Simulations
Source: Vet Sci. 2026 Feb 26;13(3):223. doi: 10.3390/vetsci13030223 (PMC13030654; doi:10.3390/vetsci13030223)
Supplement: Supplementary file 1 [file vetsci-13-00223-s001.zip › Supplementary data S1.pdf]

Supplementary data S1

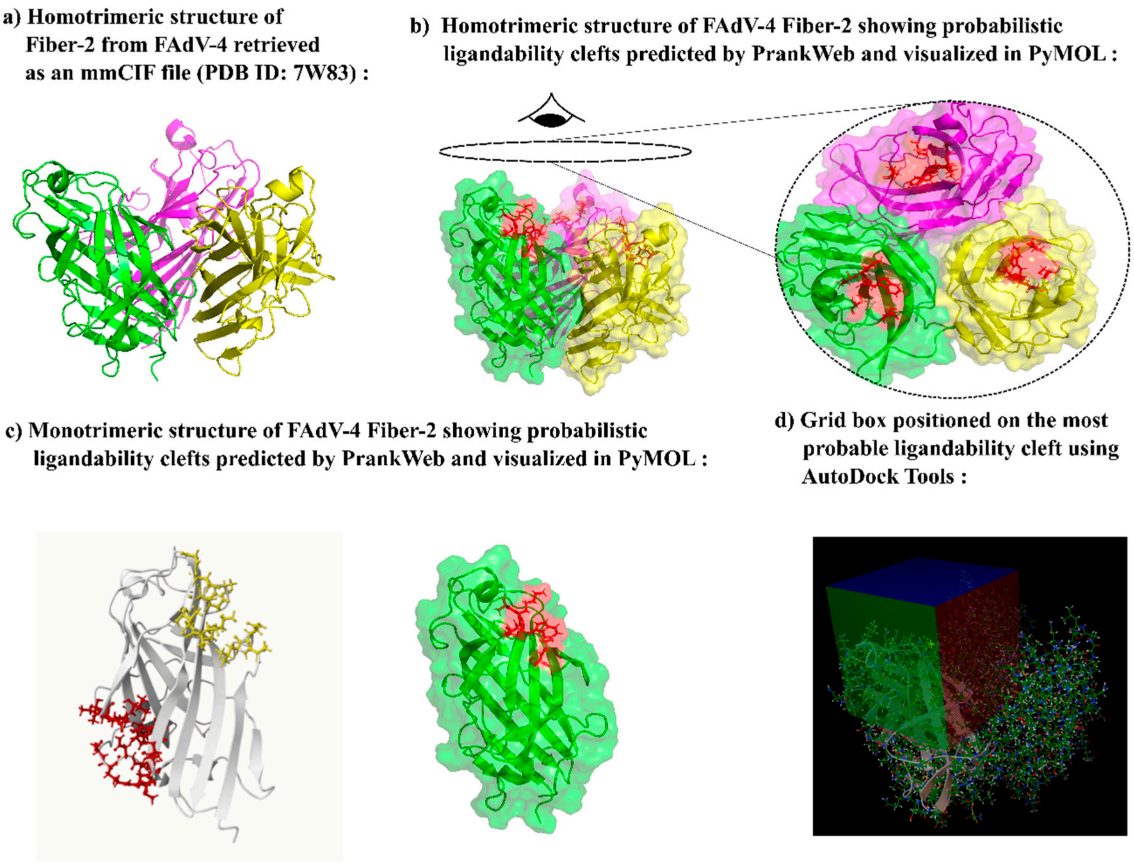

Figure S1: Computational analysis of FAdV-4’s Fiber-2 and the probabilistic ligandability cleft targeted using AutoDock Tools.

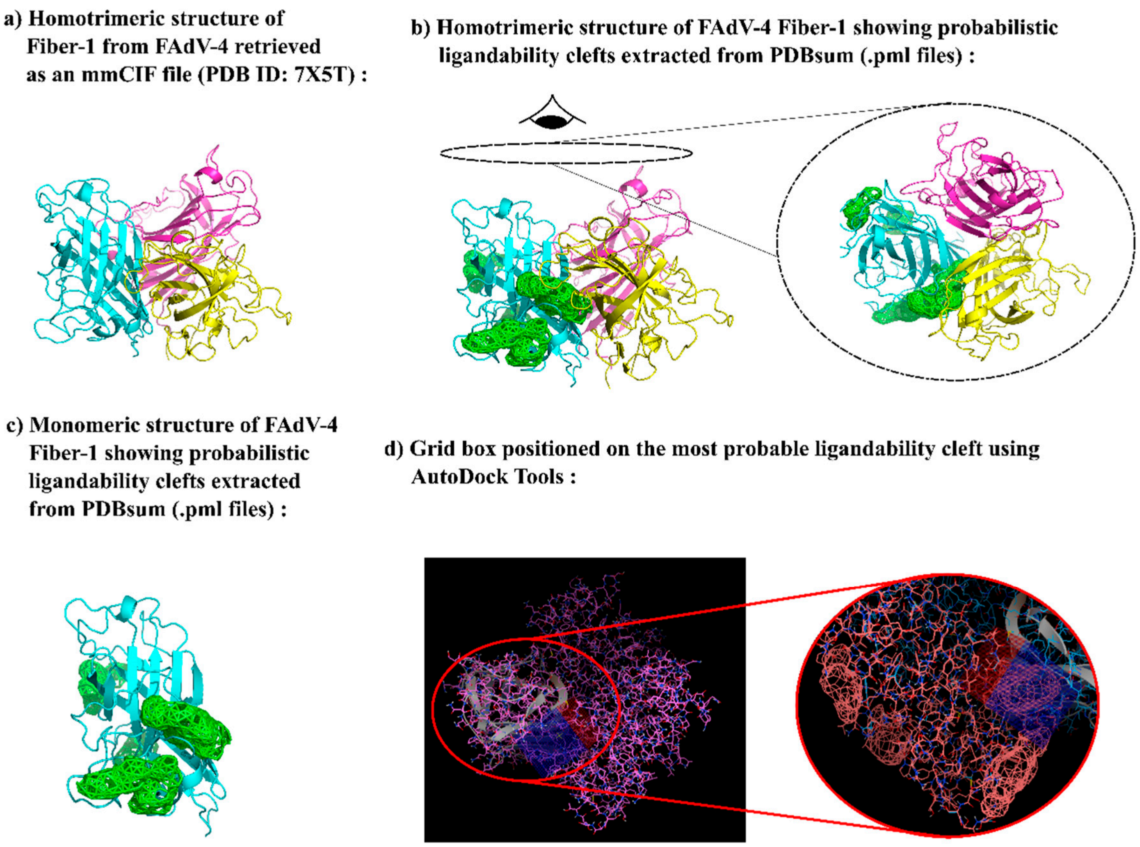

Figure S2: Computational analysis of FAdV-4's Fiber-1 and the probabilistic ligandability cleft targeted using AutoDock Tools.

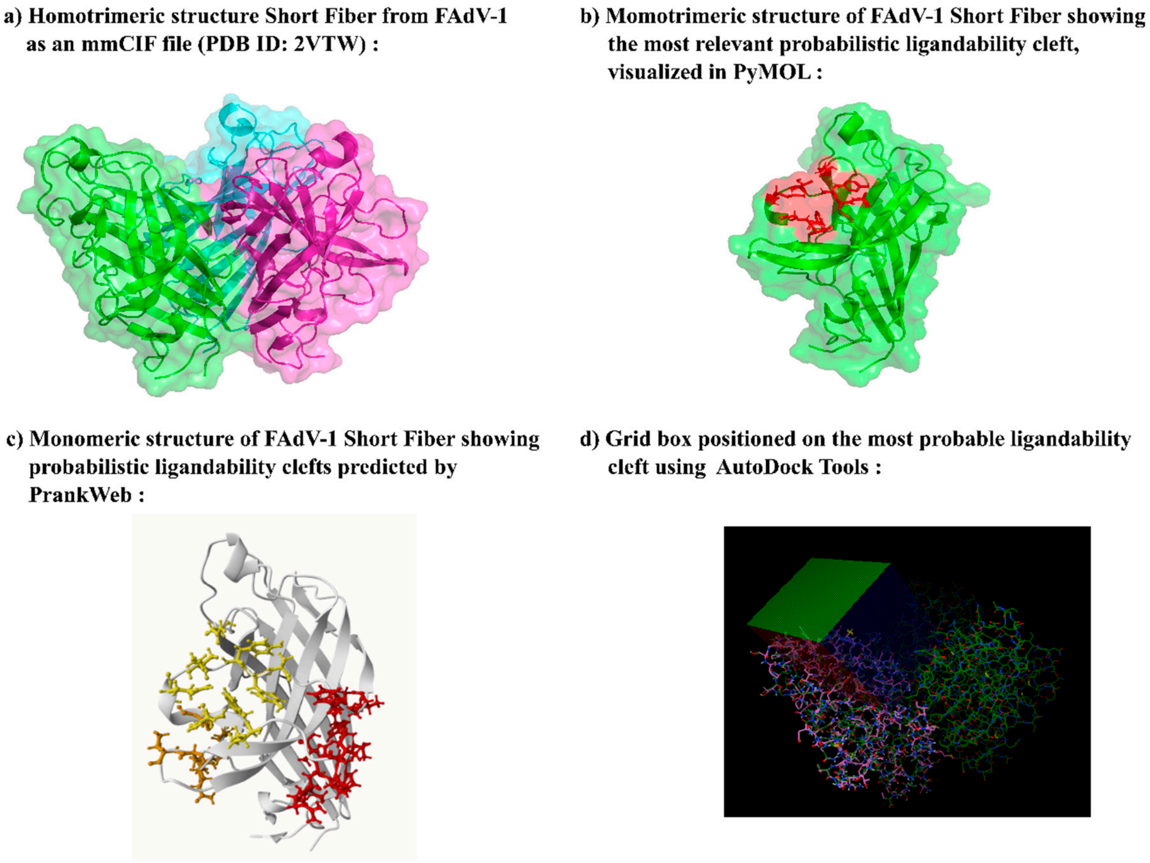

Figure S3: Computational analysis of FAdV-1's Short Fiber and the probabilistic ligandability cleft targeted using AutoDock Tools.

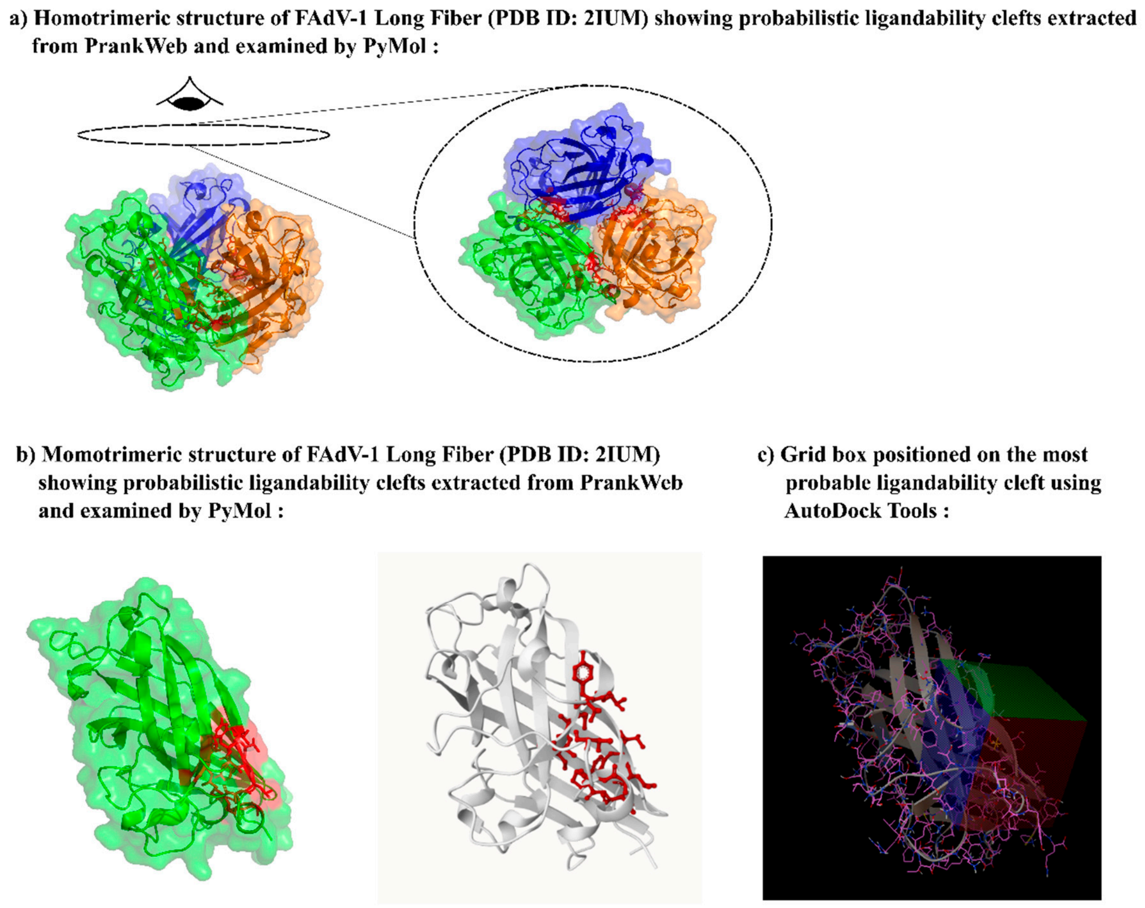

Figure S4: Computational analysis of FAdV-1's Long Fiber and the probabilistic ligandability cleft targeted using AutoDock Tools.
